# Environmental Pollution from Pharmaceuticals

**DOI:** 10.3390/life15091341

**Published:** 2025-08-25

**Authors:** Stefania Zampelli, Roberto Verna

**Affiliations:** 1 Research and Training Center for Health and Social Issues, University Guglielmo Marconi, Via Plinio, 44, 00193 Roma, Italy; stefania.zampelli@gmail.com; 2 Academy for Health and Clinical Research, Sapienza University of Rome, Piazzale Aldo Moro, 5, 00185 Roma, Italy

**Keywords:** environment, pollution, drugs

## Abstract

Pharmaceuticals are invaluable allies in human and veterinary medicine, but their indiscriminate use can transform them into formidable pollutants—a threat that remains largely underestimated. The growth of the pharmaceutical market has increased the environmental presence of drugs, both as original compounds during production and as metabolites following administration. Particularly concerning is the presence of antibiotics in wastewater, which contributes to the development and spread of bacterial resistance. This review analyzes the primary causes of environmental pharmaceutical pollution and its impact on ecosystems. We describe the most significant results obtained from analyzing wastewater, primarily in Italy, but also in other European countries, and examine the official positions of environmental protection organizations.

## 1. Introduction

Environmental pollution from pharmaceuticals represents an increasingly relevant but often underestimated problem compared to other ecological issues such as climate change or microplastic contamination.

While drugs are precious allies of human health, they can also become formidable pollutants. A significant portion of pharmaceuticals ends its life cycle in the environment—in workplaces, food systems, and other settings—creating indirect risks to human health. The expansion of the pharmaceutical market has increased the environmental presence of drugs, both as original compounds and their metabolites, which may be more toxic than the parent compounds, as their ultimate targets remain unknown [1,2].

The degree of pharmaceutical transport between different environmental compartments depends primarily on a substance’s absorption characteristics in soils, sedimentation systems, water bodies, and treatment plants, which varies considerably among different pharmaceutical products. The presence of antibiotics in wastewater contributes to the development and spread of bacterial resistance while altering environmental microbiota. In veterinary applications, drugs and metabolites can be detected at high concentrations in meat, fish, and eggs. Maximum Residue Levels (MRLs) monitor their presence to protect not only animal health, but also food safety.

The sources of pharmaceutical products in the environment encompass all phases from production through human and/or animal use to disposal. A survey conducted across various European countries provided detailed information on dispersion methods (Table 1). According to the European Union’s strategic approach regarding the environmental impact of pharmaceuticals (Brussels, 11.3.2019, COM, 2019), environmental release occurs through the following:

- Discharge of effluents from urban wastewater treatment plants containing pharmaceuticals and unused medications improperly disposed of in toilets and sinks, despite the existence of proper collection systems;
- Spreading of livestock effluents;
- Aquaculture, where pharmaceuticals are often administered with feed.

Additional sources include the following:

- Discharge of effluents from manufacturing plants (particularly outside the European Union);
- Spreading of sewage sludge containing drugs eliminated from wastewater;
- Livestock grazing;
- Pet care;
- Improper disposal of unused drugs and contaminated waste in landfills.

The most concerning phase is drug consumption and its subsequent release through excretions and incorrect disposal. Between 30% and 90% of orally administered doses are excreted in urine; consequently, the greater the quantity of a drug assimilated, the greater its environmental presence. Once excreted, a drug and its metabolites enter the sewage system and are released into surface water [3].

Hospital wastewater represents another source of drug pollution. It is believed that even after death, substances administered during the final stages of human or animal life can be released into groundwater at cemeteries or other burial sites. Furthermore, all veterinary medicines follow the same elimination pathways as human medications. For grazing animals particularly, drugs are found directly in irrigation waters and soil. While it is currently impossible to quantify the damage caused by drugs that indirectly reach humans, the most significant harm appears to be occurring to ecosystems.

## 2. Impact on Ecosystems

Pharmaceutical products in the environment represent an emerging threat to marine ecosystems. In Italy, the drugs most frequently found in wastewater belong to the nonsteroidal anti-inflammatory drug category, which are highly harmful to ecosystems, causing cellular damage to fish with adverse effects on respiration, growth, and reproductive capacity [4]. Researchers have therefore focused primarily on marine ecosystems, as many contaminants produced by human activities converge in rivers, estuaries, and coastal waters, with virtually no marine ecosystem remaining unaffected.

Original pharmaceutical products or their metabolites have been detected in various environmental compartments, explaining researchers’ heightened attention to various marine animal species. Of particular interest are recent studies demonstrating behavioral alterations in fish due to antipsychotic drugs. These phenomena occur because these animals share many neurotransmitters with humans. Monoamines such as serotonin, dopamine, and norepinephrine are found in vertebrates as well as many invertebrates including insects, amphibians, and fish [5]. As human drugs are released almost continuously into the environment, wild organisms are exposed for much longer periods than those used in standard laboratory tests (Table 1).

**Table 1:** Examples of drugs found in aquatic species.

Pharmacological Class
Analgesics
Antibiotics
Antidepressants
Antidiabetics
Antiepileptics
Antihypertensives
Anti-Inflammatories
Antineoplastics
Antipsychotics
Antivirals

Source. Examples of pharmacological classes found in aquatic species (modified from Carter et al., 2019) [6].

Researchers have begun examining effects caused by long-term, low-level exposure to pharmaceuticals. Currently, determining the exact toxicological risk in various ecosystems remains difficult; further research is needed to consider multiple variables such as different bioaccumulation mechanisms (influenced by pH, oxygen concentration, and salinity), the role of contaminant mixtures, the physicochemical characteristics of various natural environments, and the biological characteristics of their inhabitants [7].

Some aquatic species contain high concentrations of ethinylestradiol (EE2), which interferes with species reproduction. EE2 is a synthetic estrogen used primarily in oral contraceptives. Its release sources include the following:

- EE2 excretion in urine from individuals taking oral contraceptives; wastewater treatment plants cannot always eliminate it completely;
- Entry into rivers, lakes, and eventually oceans, causing several negative effects on marine ecosystems;
- Hormonal dysfunctions, as EE2 acts as an endocrine disruptor, altering the hormonal systems of aquatic organisms;
- Feminization of male fish, leading to abnormal production of female-typical proteins (such as vitellogenin) and, in some cases, reduced fertility.

These effects alter reproduction by reducing population numbers within species, thereby influencing the marine food chain (Table 2).

## 3. Risks to Human Health

For humans, the risk associated with consuming contaminated drinking water appears minimal. Assuming a daily intake of 2 L of drug-contaminated water for 70 years, lifetime consumption would still remain below a single therapeutic dose. However, continued exposure over time through water and the food chain should not be underestimated. In Europe, contaminant levels vary by country depending on

- Fertilizer usage quantities;
- Drinking water treatment methods;
- Drug exposure concentrations.

An example of this indirect exposure occurs with organic fertilizers, which transport medicines into food, causing indirect antibiotic exposure to human intestinal flora and contributing to one of our era’s greatest challenges: antibiotic resistance.

Studies of hospital and municipal purification system effluents have revealed that ideal platforms are often created for coexistence and interaction among antibiotics, bacteria, and resistance genes. These genes can be transmitted horizontally between bacteria through conjugation, transduction, or transformation mechanisms [9].

This creates a “cascade diffusion” problem, in which resistant genes are transported from individual to individual. In agriculture, the same problem can arise when contaminated manure or sludge is used, absorbed by soil, and transferred to plant roots and leaves.

## 4. Studies and Results

Pharmaceuticals are not currently included in European legislation regulating priority substances in the water sector [10]. However, some pharmaceuticals belonging to estrogens, antibiotics, and painkiller classes have recently been included in a watch list of substances to be monitored in the European Union to evaluate their possible inclusion in priority substance lists [11].

Drugs were first monitored in Italian surface and drinking water in 2000 [12], and subsequent studies have evaluated their presence in major watercourses [13,14] and their removal in purification plants, identifying the most persistent substances [15].

Generally, in rivers, substances detected at higher concentrations are those more stable during water treatment by purification plants.

A 2005 study by Ettore Zuccato et al. [16] identified environmental drugs and provided an overall contamination level assessment, enabling monitoring to focus on molecules most likely to be considered problematic. That study refined the list by analyzing pharmaceutical products in wastewater treatment plants (STPs) and comparing their concentrations with previously measured surface water levels.

This approach identified a limited group of priority pollutants in Italy’s aquatic environment, including the following:

- Ofloxacin
- Furosemide
- Atenolol
- Hydrochlorothiazide
- Carbamazepine
- Ibuprofen
- Spiramycin
- Bezafibrate
- Erythromycin
- Lincomycin
- Clarithromycin

In 2018, the same research group studied the presence and distribution of numerous emerging contaminants, including drugs, throughout the entire integrated water cycle in Italy’s most urbanized and industrialized area—the Lambro river basin [17].

The study monitored 37 drugs belonging to 11 different therapeutic classes in wastewater, surface water, groundwater, and drinking water, quantified their environmental release through water purifiers and other widespread sources, and studied their environmental fate.

Table 3 shows drug concentrations measured in wastewater entering and leaving three Milan purification plants (concentrations > 50 ng/L entering) and in surface water.

Of the 37 substances analyzed, 20 were consistently present in wastewater and surface water. The most abundant substances in untreated water were painkillers and atenolol, measured at concentrations above 1 μg/L, with various antibiotics (clarithromycin, ciprofloxacin, and ofloxacin), lipid-lowering drugs (bezafibrate), diuretics (hydrochlorothiazide and furosemide), and the antiepileptic carbamazepine also present at levels above 0.5 μg/L.

Most substances remain in outgoing water and rivers, although generally at lower levels. These concentrations depend directly on purifier removal capacity, which varies significantly based on the substance’s chemical/physical nature.

Most painkillers, especially paracetamol and ibuprofen, are removed very effectively, while diuretics, diclofenac (painkiller), and some antibiotics, such as dehydro-erythromycin and clarithromycin, are more stable.

## 5. Risk Scenarios

The described study highlighted possible risk scenarios, evaluating environmental impacts from surface water contamination [18] and human impacts from drinking water consumption [19].

Based on current assessments, there is no human risk given, the low pharmaceutical presence in drinking water, while potential environmental threats are highlighted, with risks exceeding acceptable thresholds in all analyzed rivers.

A particularly critical scenario involves the ubiquitous presence of antibiotics in the environment. As previously mentioned, this implies a risk of resistance transmission from anthropized environments to human pathogens and a consequent reduction in positive effects from targeted clinical and veterinary actions. Antibiotic resistance currently represents one of the main public health problems; therefore, understanding factors that promote it is essential.

## 6. Possible Non-Legislative Actions

To reduce environmental contamination from pharmaceuticals without resorting to new binding regulations, several non-legislative actions can be implemented, including

- Developing and harmonizing unused medicine collection systems;
- Strengthening source separation and wastewater treatment measures;
- Disseminating information and educating the population;
- Making medicines more environmentally friendly.

Pharmaceuticals can undergo biotic or abiotic degradation in soil and water, reducing their potency, although some degradation products remain dangerous.

“Biotic” factors refer to living ecosystem components, including all organisms such as plants, animals, fungi, and microorganisms. In contrast, abiotic factors are non-living ecosystem components, including physical and chemical environmental elements that directly influence life but are not products of biological activity: water, sunlight, temperature, soil, and wind.

Degradation varies significantly depending on chemistry, biology, and climatic conditions. For example, the antiparasitic ivermectin has a half-life six times longer in winter than in summer conditions, and the compound degrades faster in sandy soils than in sandy clay soils. This increases problem complexity and requires individual solutions for specific pharmaceuticals and applications.

Attention has also been focused on fat-soluble drugs, which accumulate in animal adipose tissue as they do in humans.

Studies have shown that drug exposure can lead to serious ecotoxicological effects. An example is the sharp decline in vulture populations on the Indian subcontinent. The primary cause is linked to diclofenac poisoning: vultures were exposed to the drug by feeding on cattle carcasses previously treated with the drug, and subsequently died of kidney failure [20].

A thorough knowledge of drugs, including their structure and mode of action, would help focus environmental assessments and improve understanding of how drugs interact with the environment and animal species.

## 7. Legislative Factors

The community approach to assessing and controlling environmental risks from medicinal products for human and veterinary use throughout their life cycle requires the revision of numerous legislative instruments.

The European Commission published a document at the end of 2020 [21] proposing a new strategy to reduce environmental impact based on the following five key points:

- Innovate to obtain safe and sustainable chemicals in the EU;
- Strengthen the EU legal framework to address urgent environmental and health concerns;
- Simplify and consolidate measures to improve the legal framework;
- Build a comprehensive knowledge base regarding chemicals;
- Set an example for good global chemical management.

At the technological level, extensive research is underway to establish the most effective and economically sustainable purification systems [22].

At the research level, forming multidisciplinary research groups, establishing shared experimental models (allowing comparison between various research results), and sharing risk calculation methods for both the environment and human health would be beneficial.

## 8. Ecopharmacovigilance (EPV)

The continuous and rapid development of the global pharmaceutical industry and increased consumption of drugs for human and veterinary use [23] require maximum attention to drug impacts on the environment, animals, and humans. Furthermore, they require constant commitment from competent authorities to protect health by defining increasingly effective strategies for monitoring, minimizing, and preventing drug pollution.

These issues are addressed by Ecopharmacovigilance (EPV), an emerging science that includes, according to the World Health Organization definition, “the activities of detection, evaluation, understanding, and prevention of negative effects linked to the presence of pharmaceutical products in the environment.”

The principles inspiring Ecopharmacovigilance have become, especially in the West, an integral part of regulatory legislation governing drug research, production, development, and disposal, thanks to growing awareness and sensitivity toward this topic of great relevance for planetary health.

EPV approaches include green drug design, green (or sustainable) chemistry, biodegradable product development, industrial emission minimization, education on rational drug use, prescription practice improvement, and unused drug management and disposal. These new approaches have been introduced in the environmental monitoring of antidepressants, antibacterials such as fluoroquinolones, hormones, paracetamol, and diclofenac.

## 9. Environmental Risk Assessment

The term ERA (Environmental Risk Assessment) refers to the process used to analyze and predict the potentially harmful effects of chemicals, biological agents, or human activities on ecosystems and the environment. Assessment is not required for all drugs for human or veterinary use; weight is given to environmental risks found in the marketing authorization procedure.

The following two important directives address this subject:

- Article 8 Directive 2001/83/EC;
- Article 12 Directive 2001/83/EC.

Both focus on safety measures for all drug life processes, administration to animals and humans, waste disposal, and indications of potential risks the drug could present for environmental, human, and animal health, and plants. The entire process is analyzed case-by-case.

For example, the following veterinary drugs may not be subject to environmental information obligations:

- Electrolytes, peptides, proteins, vitamins, and other compounds naturally present in the environment;
- Companion animal medicines, and medicines for minor species bred and treated similarly to major species with existing environmental risk assessments;
- Medicines used to treat small numbers of animals within flocks or herds.

## 10. Union Policy

European Union legislation on medicinal products is the main instrument for ensuring the quality, efficacy, and safety of medicinal products for human and animal use, as well as environmental safety.

Through EU Regulation 2019/6 [24], environmental risk assessment is now mandatory for all marketing authorization applications for medicinal products for human and animal use, considering benefit/risk ratios.

Given that legislative acts have only recently been adopted, environmental risks remain.

On 10 April 2024, the EU Parliament definitively approved a directive proposal to replace historic Directive 1999/271/EC on urban wastewater treatment, implemented in Italy by Legislative Decree 152/2006. Among the main innovations of the proposed directive, which must also be approved by the EU Council, is a new “roadmap” for urban wastewater treatment obligations based on the equivalent population served.

Specific innovations are as follows:

- All agglomerations serving at least 1000 population equivalents (p.e.) must have secondary treatment for biodegradable pollutant removal by 2035;
- All agglomerations serving at least 150,000 p.e. must have tertiary treatment for nitrogen and phosphorus removal by 2039;
- All agglomerations serving at least 150,000 p.e., and in some cases more than 10,000 p.e., must have quaternary treatment for certain micropollutant removal by 2045.

Costs for this latter treatment should be primarily borne by manufacturing companies, based on extended producer responsibility principles, which presents significant implementation challenges.

## 11. Water Safety Plan

When discussing water supplied for consumption, it is important to emphasize the importance of WSPs (Water Safety Plans). In 2004, the WHO introduced this model to ensure drinking water system safety, supplied water quality, and consumer health protection. This became mandatory in Italy in 2017.

WSPs remain the most effective means of ensuring the long-term safety and quality of water supplied for consumption. Advanced wastewater treatment methods include the following [25]:

- **Physical methods:** Various filter types (sand, membrane, etc.) can remove particle-bound medicines through different processes, such as reverse osmosis, depending on membrane pore diameter;
- **Chemical methods:** Advanced oxidation processes with specific reagents used to oxidize medicines.

## 12. Watch List

The second biennial watch list update was published in 2020, and lists substances selected for monitoring that could represent significant European Union aquatic environmental dangers, but for which scientifically reliable data on actual environmental and citizen risks are not yet available. Periodic list updates are necessary due to the continuous introduction of new synthetic molecules to the market, requiring environmental control bodies to provide prompt and flexible responses to emerging pollutant challenges. Including several pharmaceutical products on the list serves the European Union’s desire to control pharmaceutical environmental impact.

Commission Implementing Decision (EU) 2020/1161 of 4 August 2020 established a watch list of substances to be monitored at Union level in water policy pursuant to Directive 2008/105/EC of the European Parliament and of the Council [10].

The European Commission, with regard to the Treaty on the Functioning of the European Union, determined that watch list substances must be selected from those that, according to available information, could present significant Union-level risks to or via the aquatic environment, but for which insufficient monitoring data prevents conclusions about actual risks.

The first substance watch list was established by Commission Implementing Decision (EU) 2015/495 and contained ten substances or substance groups, as shown in the table below (Table 4).

## 13. Analytical Strategies for the Identification of Pharmaceutical Products

Investigation methodologies are used to separate, identify, and quantify the residues and metabolites of medicinal products for human and veterinary use.

The most widely used methodology is liquid chromatography, which is used for approximately 40% of tests. Biochemical tests follow, with 15% usage.

The following methods are used in smaller percentages:

- Mass spectrometry (4%);
- Capillary electrophoresis (3%);
- Gas chromatography (2%);
- Electrochemical methods (1.2%).

## 14. Conclusions

The problem of pharmaceutical abuse and improper disposal represents a challenge for both public health and the environment.

Excessive production and indiscriminate use lead to chemical accumulation that, if not properly managed, can contaminate soil and water, causing ecosystem damage and potential human health risks.

To address this problem, promoting greater awareness regarding pharmaceutical use and disposal is essential.

Best practices include the following:

- **Avoid accumulation and improper use:** Purchase only necessary medicines and avoid self-medication to reduce waste risk and drug resistance, especially antibiotic resistance [16];
- **Dispose of expired or unused medicines correctly:** Do not dispose of them in sinks or regular waste; place them in appropriate containers available at pharmacies, which ensure safe disposal;
- **Support research into greener medicines:** Promote the development of medicines with lower environmental impact, both in composition and packaging;
- **Spread best practices:** Raise population awareness through educational campaigns involving schools, pharmacies, and health facilities.

Each of us can contribute to reducing pharmaceutical pollution through small daily actions that help protect the environment and ensure a more responsible use of medical resources.

## Figures and Tables

**Table 2:** Pharmaceutical substances and their ecosystem effects.

Substance	Class	Effect
Fenfluramine	Anorectic	Enhances serotonin (5-HT) release in shrimp, triggering ovarian stimulating hormone release, resulting in increased oocyte and vitelline quantities in fiddler crabs; stimulates gonad stimulating hormone production, accelerating testicular maturation
17α-ethinyl estradiol	Synthetic steroid	Endocrine interference effects on fish, reptiles, and invertebrates
Methyltestosterone	Synthetic steroid	Intersex conditions, reduced fecundity, altered oogenesis and spermatogenesis in snails
Avermectins	Antiparasitic	**Adult insects:** water balance loss, feeding disruption, reduced fat accumulation, delayed ovarian development, decreased fecundity, impaired mating. **Juvenile insects:** developmental delay, reduced growth rates, physical abnormalities, impaired pupation or emergence, loss of developmental symmetry
Tetracyclines, Macrolides, Streptomycin	Antibacterial	Antibacterial resistance measured in soil bacteria from pig slurry-treated sites
Cypermethrin	Ectoparasiticide	Impact on manure decomposition
Fenbendazole	Antiparasitic	Impact on manure decomposition
Tylosin	Antibacterial	Impact on soil microbial community digestion
Erythromycin	Antibacterial	Growth inhibition in cyanobacteria and aquatic plants
Tetracycline	Antibacterial	Growth inhibition in cyanobacteria and aquatic plants
Ibuprofen	Anti-inflammatory	Growth stimulation in cyanobacteria and growth inhibition in aquatic plants
Fenofibrate	Lipid regulator	Inhibition of basal EROD activity in rainbow trout hepatocyte cultures
Carbamazepine	Analgesic	Inhibition of basal EROD activity in rainbow trout hepatocyte cultures, inhibition of *Chironomus riparius* emergence
Diclofenac	Analgesic	Inhibition of basal EROD activity in rainbow trout hepatocyte cultures

Source. Examples of pharmaceutical substances and their ecosystem effects (modified from Boxall ABA, 2004) [8].

**Table 3:** Drug concentrations in Milan wastewater treatment plants and surface water.

Drug	Average Purifier Concentrations (IN) ng/L	Average Purifier Concentrations (OUT) ng/L	Average Surface Water Concentrations ng/L
Paracetamol	2715	8	17
Atenolol	1682	526	220
Ibuprofen	1406	60	98
Naproxen	1057	209	86
Ketoprofen	1011	173	14
Clarithromycin	960	466	195
Bezafibrate	829	457	46
Diclofenac	675	469	260
Carbamazepine	636	245	121
Ciprofloxacin	618	202	34
Ofloxacin	546	294	97
Hydrochlorothiazide	531	349	174
Furosemide	508	554	59
Dehydro-erythromycin	269	202	62
Gemfibrozil	211	23	16
Ranitidine	115	78	8
Sulfamethoxazole	90	75	7
Enalapril	87	12	5
Vancomycin	65	22	8
Atorvastatin	64	11	2

Mean concentrations (ng/L) measured in wastewater entering (IN) and exiting (OUT) three purification plants in Milan and in surface water (Lambro, Seveso, and Olona rivers) [18].

**Table 4:** Watch list of substances to be monitored at Union level.

Name of Substance or Group	CAS Number (1)	EU Number (2)	Analysis Methods (3), (4)	Maximum Detection Limit (ng/L)
Metaflumizone	139968-49-3	604-167-6	LLE-LC-MS-MS or SPE-LC-MS-MS	65
Amoxicillin				78
Ciprofloxacin				89
Sulfamethoxazole (5)				100
Trimethoprim (5)				100
Venlafaxine and O-desmethylvenlafaxine (6)				6
**Azole compounds: (7)**				
Clotrimazole	23593-75-1	245-764-8		20
Fluconazole	86386-73-4	627-806-0		250
Imazalil	35554-44-0	252-615-0		800
Ipconazole	125225-28-7	603-038-1		44
Metconazole	125116-23-6	603-031-3		29
Penconazole	66246-88-6	245-324-5		200
Prochloraz	67747-09-5	266-276-6		1700
Tebuconazole	107534-96-3	266-994-5		161
Tetraconazole	112281-77-3	403-640-2		240
		407-760-6		1900
Dimoxystrobin	149961-32-4	604-712-8	SPE-LC-MS-MS	32
Famoxadone	131807-57-3	603-520-1	SPE-LC-MS-MS	8.5

**Legend:** (1) Chemical Abstracts Services; (2) European Union Number; (3) To ensure comparability of results from different Member States, all substances are monitored in whole water samples; (4) Extraction methods: LLE-Liquid-Liquid Extraction; SPE-Solid Phase Extraction Analytical methods: LC-MS-MS-Liquid Chromatography, Triple Quadrupole Tandem Mass Spectrometry; (5) Sulfamethoxazole and trimethoprim are analyzed together in the same samples, but reported as individual concentrations; (6) Venlafaxine and O-desmethylvenlafaxine are analyzed together in the same samples, but reported as individual concentrations; (7) Azole compounds are analyzed together in the same samples, but reported as individual concentrations.
